# Subjective language disturbances in young patients at ultra-high risk for psychosis (UHR): what relevance for clinical prognosis? A 2-year follow-up study

**DOI:** 10.1007/s00406-025-02094-w

**Published:** 2025-08-28

**Authors:** Lorenzo Pelizza, Andrea Berti, Alessandro Di Lisi, Michele La Maida, Emanuela Leuci, Emanuela Quattrone, Derna Palmisano, Simona Pupo, Giuseppina Paulillo, Clara Pellegrini, Pietro Pellegrini, Marco Menchetti

**Affiliations:** 1 Department of Biomedical and Neuromotor Sciences, Alma Mater Studiorum – Università di Bologna, Bologna (BO), Italy; 2 Department of Mental Health and Pathological Addiction, Azienda USL di Parma, Parma, PR Italy; 3 Department of Neuroscience, Rehabilitation, Ophthalmology, Genetics, Maternal and Child Health (DINOGMI), Section of Psychiatry, University of Genoa, Genoa, Italy; 4 Pain Therapy Service, Department of Medicine and Surgery, Azienda Ospedaliero-Universitaria di Parma, Parma, PR Italy; 5 Istituto di Psichiatria “Paolo Ottonello” - viale Pepoli, Bologna (BO), 5 - 40126 Italy

**Keywords:** Language disorder, Ultra-High risk, Basic symptoms, Early intervention in psychosis, Outcome, Formal thought disorder

## Abstract

**Introduction:**

Language impairment has the potential to predict the onset and progression of psychosis. However, it was mainly examined using automated extraction of quantitative linguistic features and their associations with observable psychopathological aspects of psychosis (e.g., formal thought disorders). Little interest has been paid to subjective language disturbances that should phenomenologically anticipate these more objective clinical features. Therefore, the aim of this examination was to investigate subjective language disorders in a Ultra-High Risk (UHR) sample and their associations with clinical and functional outcomes along 2 years of follow-up.

**Methods:**

170 UHR participants (88 [51.8%] females; mean age = 19.52 ± 6.03 years) were assessed for a broad range of clinical outcomes, including psychosis transition, clinical and functional remission measured with the Positive and Negative Syndrome Scale and the Social and Occupational Functioning Assessment Scale. Comparisons between patients with or without baseline subjective language disorders (specifically explored with the Schizophrenia Proneness Instrument) were analyzed using Chi-Square and Mann-Whitney tests, Kaplan-Meier survival analysis, and binary logistic regression analysis.

**Results:**

Across the follow-up, the UHR subgroup with language disorders at entry (*n* = 80) showed higher and more enduring severity in psychopathology (especially negative and disorganized features), as well as poorer socio-occupational functioning over time.

**Conclusion:**

The presence of subjective language disturbances at baseline identifies a subgroup of UHR youths with poorer psychopathological and functional prognosis. Further studies examining their association with quantitative linguistic biomarkers are needed, especially to better predict the onset and progression of psychosis.

**Supplementary Information:**

The online version contains supplementary material available at 10.1007/s00406-025-02094-w.

## Introduction

The *Clinical High-Risk for Psychosis* status is known to be a conceptual synthesis from the theoretical model of “Ultra-High-Risk” (UHR) mental states [1] and the “Basic Symptoms” (BS) construct [2], heritage of the Bonn research group in Germany [3]. Research on these risk states for psychosis has been carried forward to reach a deeper knowledge on etiological/pathogenic patterns of illness development and to define which at-risk subgroup has a greater chance to transition to psychotic disorder [4, 5]. This remarkable action in public mental healthcare finds its roots in the opportunity to identify and treat help-seeking individuals before crossing the psychosis threshold, in order to prevent/reduce the risk of conversion or at least, for whom going forward to transition, to longitudinally ensure a lower impact of illness and a better functioning in everyday life, thanks to early multidisciplinary interventions that collected evidence through time [6, 7].

*Language disorders* represent a fundamental chapter in the comprehensive assessment of UHR patients, especially as psychopathological precursors in the early detection of psychosis [8, 9]. Therefore, also in schizophrenia proneness instrument we can find items that investigate speech and language disturbances in a *subjective* perspective, in terms of both expression and reception [10]. In this respect, current research on early psychosis recently focused on mainly identifying objective language abnormalities (such as impairment in prosody, syntactic structure, and semantic coherence during Natural Language Processing [NLP]) [11, 12]. Moreover, automated speech analysis systems showed some efficacy in predicting transition to psychosis in UHR cohorts, although such studies were conducted on small sample sizes [13]. Furthermore, Rezaii and co-workers [14] correlated semantic density with positive symptoms, while speaking voices with negative symptoms, suggesting that two variables with such association had high predictive power with respect to psychosis conversion in prodromal patients. In this respect, the role of language disturbances, especially in Schizophrenia Spectrum Disorders, has been debated for a long time, given the intimate correlation between language and thought disorders [15–18]. More recently, specifically exploring clinical markers of early psychosis from language process, some authors used advanced linguistic analysis models (such as speech graph, vector unpacking, and latent complex analysis) and language-related abnormalities in neuroimaging, to detect the most subtle disturbances in the speech flow of patients at the psychosis onset [19].

However, language disturbances and formal thought disorders as well are trans-diagnostic symptoms, occurring not only in SSD but also in patients with affective disorders. Specifically, SSD patients have more idiosyncratic speech and nonspecific disorganization than patients with affective disorders. Discriminating between SSD and affective disorders looks to be helpful, given the reduced connectivity score got from speech graph models [19, 20].

Within UHR samples, a latent analysis model by DeVylder and colleagues [21] showed an increased hazard risk for psychosis onset in those at-risk patients with disordered communication at baseline. More recently, Bianciardi and co-workers [22] ruled out specific temporal (e.g., articulation rate, speech rate, pause rate, average syllable and pause duration) and prosodic features of language (e.g., glottal pulse period, jitter, shimmer, Harmonics-to-Noise Ratio [HNR], Noise-to Harmonics Ratio [NHR], voice breaks and unvoiced frames) from being markers of early psychosis using an automated acoustic analysis. Bilgrami and colleagues [23] also showed positive correlations between NLP metric of coherence and positive thought disorder severity, and between NLP metric of complexity and negative thought disorder severity. Furthermore, Meijer and co-workers [24] reported a significant correlation between semantic fluency and grey matter density in the UHR subgroup with psychosis transition. However, empirical studies investigating language and speech disturbances in at-risk patients considered exclusively objective features, also reporting relevant associations among disorganization, poverty of content, reduced referential cohesion, impaired semantic verbal fluency, and risk of psychosis conversion [19]. Little is known about the role of subjective language disturbances, especially early in the psychopathological trajectory of psychosis, although they have been included in the basic symptom criteria and in the Schizophrenia Proneness Instrument for their relevance in early detection of schizophrenia [2, 25].

To our knowledge, no previous study specifically examining subjective language disorders in UHR patients has been published in the literature to date, especially on how these self-reported symptoms are related to the risk of overcoming psychosis threshold. Therefore, the goals of this examination were: (a) to explore clinical and socio-demographic comparisons between UHR with and without subjective language disturbances at entry, and (b) to examine any inter-group longitudinal difference in terms of global functioning, psychopathological features, and outcome indicators across a 2-year follow-up period.

## Methods

### Setting and participants

All UHR participants were sequentially enrolled within the “Parma At-Risk Mental States” (*PARMS*) program from January 2016 to December 2021. Their participation in the PARMS program (and consequently in this study) was voluntary. We had no data about patients that were eligible for the PARMS protocol but refused specific “Early Intervention in Psychosis” (EIP) interventions. No comparison was possible between PARMS participants and nonparticipants. The PARMS is a specialized EIP infrastructure diffusely implemented across all adolescent and adult mental healthcare services in the Parma Department of Mental Health (Northern Italy) [26].

*Inclusion criteria* were: (a) to seek specialist mental health assistance; (b) age 12–25 years, (c) to meet UHR criteria as defined by the “Comprehensive Assessment of At-Risk Mental States” (CAARMS) [25] at the baseline assessment (i.e., Attenuated Psychotic Symptoms [APS], Brief Limited Intermittent Psychotic Symptoms [BLIPS], and Genetic Vulnerability).

*Exclusion criteria* were: (a) past overt affective or non-affective psychotic episode; (b) previous exposure to AP drug or current AP intake for a duration exceeding 4 weeks in the present illness episode, (c) known intellectual disability (IQ < 70); (d) neurological or other medical disorder with psychiatric manifestations. Past use of AP medication was considered a proxy for past psychotic episode, consistently with the original CAARMS criteria for psychosis threshold [27]. A current AP prescription of < 4 weeks was required to minimize pharmacological interference with baseline psychopathological assessment [28].

All participants and parents (if minors) provided written informed consent for their participation in the study. This research obtained approval from the local ethics committee (AVEN Ethics Committee protocol n. 559/2020/OSS*/AUSLPR) and adhered to the principles outlined in the 1964 Declaration of Helsinki and its later amendments.

### Instruments

The psychopathological evaluation encompassed the CAARMS, the Health of the Nation Outcome Scale (HoNOS) [29], the Positive And Negative Syndrome Scale (PANSS) [30], the Social and Occupational Functioning Assessment Scale (SOFAS) [27], and the SPI-CY (Schizophrenia Proneness Instrument, Child and Youth version) [31]. All assessment instruments were administered by trained PARMS team members both at baseline (T0) and every 12 months during the 2-year follow-up period (T1 and T2). For all these tools, regular scoring workshops and supervision sessions were implemented to ensure good values of their inter-rater reliability.

The *CAARMS* is a clinical interview designed to explore multiple aspects of attenuated psychopathology. Its “Positive Symptoms” subscale serves as the basis for defining both UHR and psychosis criteria. Moreover, the applied CAARMS version used functional deterioration (measured with the SOFAS) as inclusion requirement. CAARMS interviews were conducted by trained PARMS team members using the approved Italian version (CAARMS-ITA) [32, 33].

The *HoNOS* assesses mental health and social functioning in patients with severe mental illness [34], including early psychosis [35]. It was divided into four main domains: “Behavioral Problems”, “Impairment”, “Psychiatric Symptoms”, and “Social Problems” [36] A score of ≤ 2 on HoNOS items 9, 10, and 11 within the “Social Problems” domain indicated functional remission [37].

The *PANSS* is a widely used interview for assessing psychopathology in psychosis [38], also in young patients at illness onset [39]. As proposed by Shafer and Dazzi [40], we considered five key psychopathological dimensions: “Disorganization”, “Negative Symptoms”, “Positive Symptoms”, “Resistance/Excitement-activity”, and “Affect” (“Depression-Anxiety”). Symptomatic remission is indicated by a score of ≤ 3 on the 8 PANSS items specified by the “Remission in Schizophrenia Working Group’s criteria” [41].

The *SOFAS* (Social and Occupational Functioning Assessment Scale) is a related measure that specifically evaluates social and occupational functioning while disregarding symptom severity. Unlike the GAF, which considers both symptom burden and functional impairment, the SOFAS provides a purer measure of functional capacity, particularly in patients with early psychosis [27]. It is included in the CAARMS interview [42]. A SOFAS score of ≥ 60 was used as further index of functional remission [43].

The *SPI-CY* is a structured clinical interview designed to assess basic symptoms associated with an increased risk of psychosis in adolescents and young adults. The SPI-CY evaluates self-experienced disturbances across cognitive, perceptual, and affective domains that may precede the onset of psychotic disorder, helping to identify at-risk mental state populations [44]. In this study, the presence of language disorders was assessed using two items from the SPI-CY: i.e., the items C4 “Disturbance of receptive speech” and C5 “Disturbance of expressive speech” (C.1.7.). These items evaluate impairments in understanding spoken language and difficulties in verbal expression, respectively. A language disorder was identified when at least one of these two items was rated as present (i.e., with a score of ≥ 2). Furthermore, according to the authors [25, 31], a SPI-CY rating of 7 was treated as identifying trait-like features and was not considered in the language basic symptom score. As for the role of disturbances of expressive and of receptive speech in BS criteria, it should be noted that the “presence” of these symptoms as defined in this investigation did not necessarily correspond to the scores needed to satisfy the BS criteria (i.e., a score of at least 3). Indeed, the main goal of this research was to explore the presence of subjective language disorders regardless of their role in the BS criteria.

Lastly, we completed a *sociodemographic and clinical chart*, including information on Duration of Untreated Illness (DUI), new suicide attempt, suicidal ideation, functional recovery, psychosis transition, and service disengagement (for details on their operative definitions, see Supplementary Materials [Table S1]).

### Procedures

The initial DSM-5 diagnosis was established through assessments conducted by a minimum of two trained PARMS team members using the Structured Clinical Interview for DSM-5 disorders (*SCID-5*) [45]. Subjective language disturbances were then assessed at baseline using the SPI-CY. The presence of at least one of the two SPI-CY *language items* rated as “present” was considered indicative of the presence of a subjective language disorder and was used to include UHR participants in the *UHR/L + subgroup*. The remaining patients were grouped in the UHR subsample.

Within 4 weeks from baseline assessment, UHR participants were assigned to a multi-disciplinary team including a clinical psychologist, an early rehabilitation case manager, and a psychiatrist. In accordance with current official guidelines on EIP [46, 47], AP medication use at entry should be reserved to UHR patients who (a) displayed rapid deterioration in daily functioning, (b) experienced a sudden escalation of full-blown psychotic symptoms, (c) had an immediate risk of suicide or severe violence, or (d) failed to respond to psychosocial interventions. As a first-line psychopharmacological treatment, low-dose atypical AP drug was administered. Individual psychotherapy, family psychoeducation, and case management were provided to UHR patients following specific models [48, 49]. This included at least 15 sessions of individual psychotherapy [50], 12 sessions of family psychoeducation [51], and 24 sessions of early rehabilitation coordinated by a dedicated case manager over 2 years [52].

### Statistical analysis

Collected data were examined using the Statistical Package for Social Science (SPSS) version 28.0 for Windows [53]. All tests were two-tailed, with a significance p level set at 0.05. Bonferroni correction was calculated to deal with multiple comparisons and family-wise error rates [54].

At presentation, between-group comparisons were analyzed using the Chi-Square (X^2^) test for categorical variables and the Mann-Whitney (U) test for quantitative parameters (given their non-normal distributions) [55]. As for inter-group comparisons on time-to-event outcome parameters (e.g., service disengagement, new suicide attempt), Kaplan-Meier survival analysis was used [56]. As for not time-to-event dependent variables (e.g., suicidal ideation, functional recovery) binary logistic regression analyses with the UHR subgroup as independent parameter were performed [57]. Finally, mixed-design ANOVA analyses were carried out to evaluate the temporal stability of PANSS, HoNOS, and GAF scores within and between the two UHR subsamples across the 2 years of follow-up [58].

Data on medication, psychosocial intervention, psychopathology, functioning, and outcomes were collected at baseline and every 12 months throughout the 2-year follow-up period. The study initially compared sociodemographic, clinical, and treatment parameters between the two subgroups at entry. Then, we analyzed between-group differences in specific outcome measures over time.

## Results

A total of 170 UHR patients (80 [47.0%] CHR-P/L + and 90 CHR-P/L-) were included in the study. Within the UHR/L + subgroup, 65 patients had disturbance of expressive speech, while 30 had disturbance of receptive speech. Only 19 (23.7% of the UHR subgroup) showed subjective disorders of both expressive and receptive speech. Sociodemographic and clinical characteristics of the two subgroups are detailed in the Table 1. The total sample was predominantly female (52.2%) and white (87.6%), with a mean age at entry of 19.99 ± 6.06 years. According to the CAARMS, 115 participants met APS criteria, 14 BLIPS criteria, and 41 Genetic Vulnerability (GV) criteria. Specifically, 28 of them met both APS and GV criteria, while 3 both BLIPS and GV criteria. At entry, 68.2% UHR participants had an antipsychotic prescription.

**Table 1:** Baseline sociodemographic and clinical features in the two UHR subgroups (n = 170)

Variable	UHR/L + (n = 80)	UHR/L- (n = 90)	X^2^/z	p
Gender (females)	41 (51.2%)	47 (52.2%)	0.016	0.899
Nationality (Italian)	65 (81.3%)	80 (8.9%)	1.97	0.16
Ethnic group			4.075	0.307
White	69 (86.2%)	80 (88.9%)	[-.5]	
Black	2 (2.5%)	1 (1.1%)	[.7]	
Asian	3 (3.8%)	1 (1.1%)	[.6]	
North African	5 (6.3%)	5 (5.6%)	[.1]	
Hispanic	1 (1.3%)	3 (3.3%)	[-.9]	
Age at entry	20.09 ± 7.12	19.02 ± 4.85	-0.586	0.558
Education (in years)	11.13 ± 2.21	11.59 ± 2.45	-1.097	0.272
Marital status			0.022	0.999
Single	77 (96.3%)	87 (96.9%)	[-.1]	
Married/cohabitant	3 (3.8%)	3 (3.3%)	[.1]	
Living status			1.651	0.648
Alone	3 (3.8%)	3 (3.3%)	[.1]	
Living with partner	3 (3.8%)	6 (6.7%)	[-.8]	
Living with parents	74 (92.4%)	80 (8.9%)	[.8]	
Other	0 (0.0%)	1 (1.1%)	[-.9]	
Employment status			2.087	0.354
NEET	25 (31.3%)	22 (24.4%)	[1.0]	
Student	49 (61.3%)	56 (62.2%)	[-.1]	
Employed	6 (7.5%)	12 (13.3%)	[-1.2]	
Source of referral			1.096	0.954
Primary care	26 (32.5%)	32 (35.6%)	[-.4]	
Family members	8 (10.0%)	12 (13.3%)	[-.7]	
Self-referral	12 (15.0%)	12 (13.3%)	[.3]	
Emergency services	13 (16.3%)	12 (13.3%)	[.5]	
Other mental health services	11 (13.8%)	13 (14.4%)	[-.1]	
School/social service	10 (12.5%)	9 (10.0%)	[.5]	
Baseline hospitalization	16 (20.0%)	6 (6.7%)	6.683	**0.01**
Previous specialist contact	41 (51.3%)	49 (54.4%)	0.173	0.677
Past suicide attempt	9 (11.3%)	6 (6.7%)	1.106	0.293
DUI (in weeks)	61.37 ± 57.76	68.55 ± 51.65	-1.262	0.207
Substance use (at entry)	10 (12.5%)	16 (17.8%)	0.911	0.34
CHR group				
APS	58 (72.5%)	57 (63.3%)	1.626	0.202
BLIPS	8 (10.0%)	6 (6.7%)	0.623	0.43
Genetic vulnerability	14 (17.5%)	27 (30.0%)	0.459	0.39
AP prescription	42 (52.5%)	31 (34.4%)	5.635	**0.018**
AP equivalent dose of risperidone (mg/day)	2.99 ± 2.27	2.87 ± 2.11	-0.046	0.964
AD prescription	26 (32.5%)	24 (26.7%)	0.693	0.405
BDZ prescription	20 (25.0%)	24 (26.7%)	0.061	0.804
Treatment adherence	52 (65.0%)	60 (66.7%)	0.052	0.819
PANSS scores				
Positive symptoms	11.10 ± 4.36	9.91 ± 3.43	-1.544	0.123
Negative Symptoms	20.13 ± 8.28	15.22 ± 6.27	-3.953	**0.001**
Disorganization	17.16 ± 6.60	13.32 ± 3.79	-3.881	**0.001**
Affect	15.60 ± 4.91	13.97 ± 5.34	-2.093	0.18
Resistance/excitement-activity	7.49 ± 3.35	7.00 ± 3.38	-1.163	0.245
G12 Lack of judgment/insight	2.39 ± 1.44	1.99 ± 1.38	-2.197	**0.028**
P2 Conceptual disorganization	2.33 ± 1.14	1.68 ± 0.88	-3.88	**0.001**
Total score	73.97 ± 18.95	61.41 ± 15.56	-4.362	**0.001**
SOFAS score	47.75 ± 7.39	50.39 ± 8.32	-1.858	0.063
Work/school interruption (at entry)	48 (60%)	32 (35.6%)	10.158	**0.001**
HoNOS scores				
Behavioral problems	2.54 ± 1.99	2.14 ± 1.81	-1.346	0.178
Impairment	1.51 ± 1.40	1.79 ± 1.49	-1.125	0.26
Psychiatric symptoms	9.21 ± 2.77	7.48 ± 3.33	-3.118	**0.008**
Social problems	5.84 ± 3.05	4.26 ± 2.83	-3.299	**0.004**
Total score	19.10 ± 5.91	15.67 ± 6.12	-3.846	**0.001**

*UHR* Ultra-High Risk, *UHR/L* + UHR patients with language subjective disorder, *UHR/L-* UHR patients without language subjective disorder, *NEET* Not [engaged] in Employment, Education, or Training, *Law 104/92* Italian framework law for assistance, social integration, and rights for persons with disabilities, DUI Duration of Untreated Illness, *AP* Antipsychotic, *AD* Antidepressant, BDZ Benzodiazepine, *PANSS* Positive and Negative Syndrome Scale, *SOFAS* Social and Occupational Functioning Assessment Scale, *HoNOS* Health of the Nation Outcome Scale

Mean ± standard deviation, frequencies (and percentages), Chi-square (X2) test, Mann–Whitney (z) test values are reported. Adjusted residuals values are in square brackets. Bonferroni’s corrected p values are calculated. Statistically significant p values are in bold. ^*^p < .01

### Baseline comparisons

Compared to UHR/L-, the UHR/L + subgroup showed significantly higher levels in PANSS total score, particularly in Negative and Disorganization domain subscores (Table 1). Similarly, higher levels in HoNOS total score and HoNOS “Psychiatric symptoms” and “Social problems” domain subscores were reported in UHR/L + patients, alongside lower levels of clinical insight (i.e., a higher subscore in PANSS item G12 [“Lack of judgment/insight]) and a higher prevalence rate of work/school interruption. Finally, the UHR/L + subgroup showed higher prevalence rates of baseline psychiatric hospitalization and AP prescription.

### Longitudinal comparisons

Our Kaplan-Meier survival analysis results (Table 2) revealed no statistically significant inter-group differences in 2-year incidence rates of service disengagement, new psychiatric hospital admission, new suicide attempt, and transition to psychosis.

**Table 2:** Kaplan–Meier survival analysis results: comparisons on 2-year time-to-event outcome incidence rate among the two UHR subgroups

UHR subgroup	Number of events	Cumulative proportion surviving at the time		Mean (in months) for 2-year service disengagement incidence rate			
		Estimate	SE	Estimate	SE	95% CI	
						Lower bound	Upper bound
UHR/L +	31	42.5%	.055	18.500	.742	17.046	19.954
UHR/L-	39	47.8%	.053	18.178	.689	16.828	19.528
(Overall)	70	-	-	18.329	.505	17.340	19.319
Log Rank (Mantel-Cox)				Χ2	df	p	
				.298	1	.585	

UHR subgroup	Number of events	Cumulative proportion surviving at the time		Mean (in months) for 2-year new hospitalization rate			
		Estimate	SE	Estimate	SE	95% CI	
						Lower bound	Upper bound
UHR/L +	7	10.5%	.038	23.014	.417	22.197	23.830
UHR/L-	8	10.7%	.037	22.988	.391	22.221	23.755
(Overall)	15	-	-	23.000	.275	22.461	23.539
Log Rank (Mantel-Cox)				Χ2	df	p	
				.002	1	.964	

UHR-P subgroup	Number of events	Cumulative proportion surviving at the time		Mean (in months) for 2-year new suicide attempt incidence rate			
		Estimate	SE	Estimate	SE	95% CI	
						Lower bound	Upper bound
UHR/L +	3	5.0%	.029	23.671	.281	23.121	24.222
UHR/L-	3	4.6%	.027	23.711	.247	23.226	24.196
(Overall)	6	-	-	23.692	.166	23.366	24.018
Log Rank (Mantel-Cox)				Χ2	df	p	
				.015	1	.901	

UHR subgroup	Number of events	Cumulative proportion surviving at the time		Mean (in months) for 2-year psychosis transition rate			
		Estimate	SE	Estimate	SE	95% CI	
						Lower bound	Upper bound
UHR/L +	9	13.9%	.044	22.849	.439	21.990	23.709
UHR/L-	8	11.4%	.040	23.133	.365	22.418	23.847
(Overall)	17	-	-	23.000	.274	22.464	23.536
Log Rank (Mantel-Cox)				Χ2	df	p	
				.235	1	.628	

*UHR* Ultra-High Risk, *UHR/L* + UHR patients with language subjective disorder, *UHR/L-* UHR patients without language subjective disorder, *SE* Standard Error*, 95% CI* 95% Confidence Intervals, *Log Rank* Logarithm Rank Test, *X2* Chi-Square test, *df* degrees of freedom, *p* statistical value

Significant statistical p values are in bold

Binary logistic regression analysis results (Table 3) also failed to identify statistically significant inter-group differences at both 1- and 2-year follow-ups in terms of current suicidal ideation, functional recovery, PANSS symptomatic remission, SOFAS and HoNOS functional remission.

**Table 3:** Binary logistic regression analysis results for 2-year not time-to-event outcome variables in the two UHR subgroups

Dependent variable (n = 156)	UHR/L + (n = 73)	UHR/L- (n = 83)	Statistic test				
			B (SE)	HR	95% CI		p
					Lower Higher		
1-year current suicidal ideation	19 (26.0%)	18 (21.7%)	.239 (.377)	1.271	.607	2.660	525
1-year functional recovery	49 (67.1%)	58 (70.7%)	.169 (.348)	1.184	.599	2.340	.628
1-year SOFAS functional remission	43 (58.9%)	58 (69.9%)	.482 (.337)	1.619	.836	3.136	.153
1-year HoNOS functional remission	53 (72.6%)	66 (79.5%)	.382 (.378)	1.465	.698	3.073	.312
1-year PANSS symptomatic remission	46 (63.0%)	60 (72.3%)	.426 (.345)	1.531	.779	3.010	.217
1-year CHR-P persistence	25 (34.2%)	24 (28.9%)	-.247 (.346)	.781	.397	1.538	.475

Dependent variable (n = 100)	UHR/L + (n = 50)	UHR/L- (n = 50)	Statistic test				
			B (SE)	HR	95% CI		p
					Lower Higher		
2-year current suicidal ideation	10 (20.0%)	8 (16.0%)	.272 (.523)	1.312	.471	3.660	.603
2-year functional recovery	36 (72.0%)	37 (74.0%)	.102 (.451)	1.107	.458	2.678	.822
2-year SOFAS functional remission	29 (60.4%)	35 (74.5%)	.648 (.446)	1.911	.797	4.581	.147
2-year HoNOS functional remission	39 (78.0%)	43 (86.0%)	.550 (.532)	1.733	.611	4.912	.301
2-year PANSS symptomatic remission	31 (62.0%)	38 (76.0%)	.663 (.441)	1.941	.818	4.607	.133
2-year CHR-P persistence	17 (34.0%)	10 (20.0%)	-.723 (.463)	.485	.196	.202	.118

Current suicidal ideation BPRS item 4 score of ≥ 2, Functional recovery return to work/school, SOFAS functional remission SOFAS score of ≥ 60, HoNOS functional remission HoNOS item 9, 10, and 11scores of ≤ 2, PANSS symptomatic remission PANSS item P1, P2, P3, N1, N4, N6, G5, G9 scores of ≤ 3

*UHR* Ultra-High Risk, *UHR/L* + UHR patients with language subjective disorder, *UHR/L-* UHR patients without language subjective disorder. *SOFAS* Social Occupational Functioning Assessment Scale, *HoNOS* Health of the Nation Outcome Scale, *PANSS* Positive and Negative Syndrome Scale, *B* regression coefficient, *SE* Standard Error, *HR* Hazard Ratio, *95% CI* 95% confidence intervals for HR, *p* statistical significance

Significant statistical p values are in bold. Cumulative incidence rates are reported

Our mixed-design ANOVA results across the 2-year follow-up period (Table 4) revealed a significant main effect of time for all PANSS, SOFAS, and HoNOS scores, except for PANSS “Resistance/Excitement-activity” dimension subscores, indicating general clinical and functioning improvement in the total UHR sample. Notably, group differences were observed for PANSS “Positive”, “Negative”, “Disorganization”, “Affect”, and total scores, with UHR/L + patients showing persistently higher symptom severity levels across time-points. Additionally, they also showed the same group effects in terms of HoNOS “Psychiatric symptoms”, “Social problems”, and total scores. Moreover, significant group and interaction (“time×group”) effects were found for SOFAS scores over time, suggesting that UHR/L- participants longitudinally demonstrated greater improvements in functioning compared to UHR/L + peers (see Supplementary Materials [Figure S1] for details on profile plots).

**Table 4:** Mixed-design ANOVA results: psychopathological and outcome parameters across the 2-year follow-up period in the two UHR subgroups

Variable	Time effect				Group effect				Interaction effect (time x group)			
	df	F	p	η2	df	F	p	η2	df	F	p	η2
PANSS Positive symptoms	1.50	19.924	**.001**	.169	1	6.548	**.012**	.093	1.50	.274	.689	.003
PANSS Negative symptoms	1.49	23.485	**.001**	.193	1	22.045	**.001**	.184	1.49	1.485	.237	.005
PANSS Disorganization	1.48	29.765	**.001**	.233	1	20.449	**.001**	.173	1.48	3.205	.058	.032
PANSS Affect	1.48	56.421	**.001**	.365	1	8.624	**.004**	.081	1.48	.406	.606	.004
PANSS Resistance/Excitement-activity	1.58	7.599	**.002**	.072	1	.271	.604	.003	1.58	.048	.920	.001
PANSS Total score	1.53	40.149	**.001**	.291	1	20.138	**.001**	.170	1.53	1.023	.345	.010
SOFAS score	1.59	116.354	**.001**	.558	1	10.401	**.002**	.102	1.59	3.308	**.049**	.035
HoNOS Behavioral problems	1.42	54.125	**.001**	.358	1	.352	.555	.004	1.42	.358	.626	.004
HoNOS Impairment	1.99	17.184	**.001**	.149	1	.615	.435	.006	1.99	.986	.375	.010
HoNOS Psychiatric symptoms	1.61	96.897	**.001**	.497	1	9.761	**.002**	.091	1.61	1.392	.251	.014
HoNOS Social problems	1.78	50.600	**.001**	.341	1	11.473	**.002**	.105	1.78	1.441	.240	.014
HoNOS total score	1.66	101.789	**.001**	.512	1	9.618	**.003**	.090	1.66	1.258	.282	.013

Variable	EMM (SE)	
	UHR/L +	UHR/L-
T0 PANSS Positive symptoms	11.620 (.548)	9.640 (.584)
T1 PANSS Positive symptoms	9.340 (.535)	7.880 (.535)
T2 PANSS Positive symptoms	9.140 (.548)	7.640 (.548)
T0 PANSS Negative symptoms	22.420 (1.065)	15.080 (1.065)
T1 PANSS Negative symptoms	19.080 (1.103)	13.020 (1.103)
T2 PANSS Negative symptoms	17.380 (1.008)	12.060 (1.008)
T0 PANSS Disorganization	18.820 (.813)	13.340 (.813)
T1 PANSS Disorganization	15.880 (.793)	11.460 (.793)
T2 PANSS Disorganization	14.400 (.713)	11.160 (.713)
T0 PANSS Affect	16.080 (.748)	13.760 (.748)
T1 PANSS Affect	12.560 (.604)	9.900 (.604)
T2 PANSS Affect	11.260 (.626)	9.420 (.626)
T0 PANSS Resistance/Excitement	7.600 (.469)	7.260 (.469)
T1 PANSS Resistance/Excitement	6.560 (.446)	6.380 (.446)
T2 PANSS Resistance/Excitement	6.560 (.461)	6.220 (.461)
T0 PANSS Total score	79.020 (2.586)	61.060 (2.586)
T1 PANSS Total score	65.660 (2.875)	50.480 (2.875)
T2 PANSS Total score	60.940 (3.011)	48.160 (3.011)
T0 SOFAS score	46.596 (1.136)	50.489 (1.136)
T1 SOFAS score	55.830 (1.847)	65.170 (1.847)
T2 SOFAS score	61.170 (1.864)	67.106 (1.864)
T0 HoNOS Behavioral problems	2.640 (.276)	2.347 (.279)
T1 HoNOS Behavioral problems	1.240 (.217)	1.061 (2.19)
T2 HoNOS Behavioral problems	.960 (.205)	.939 (.208)
T0 HoNOS Impairment	1.500 (.204)	1.880 (.204)
T1 HoNOS Impairment	1.100 (.188)	1.160 (.188)
T2 HoNOS Impairment	.940 (.170)	1.020 (.170)
T0 HoNOS Psychiatric symptoms	9.580 (.434)	7.660 (.434)
T1 HoNOS Psychiatric symptoms	6.500 (.471)	4.360 (.471)
T2 HoNOS Psychiatric symptoms	5.120 (.495)	3.960 (.495)
T0 HoNOS Social problems	6.320 (.429)	4.580 (.429)
T1 HoNOS Social problems	4.960 (.404)	2.840 (.404)
T2 HoNOS Social problems	3.940 (.385)	2.580 (.385)
T0 HoNOS total score	20.040 (.849)	16.510 (.858)
T1 HoNOS total score	13.800 (.981)	9.306 (.991)
T2 HoNOS total score	10.960 (1.001)	8.469 (1.011)

As all Mauchly’s tests of sphericity are statistically significant (p < 0.05), Greenhouse–Geisser corrected degrees of freedom to assess the significance of the corresponding F value are used. Statistically significant p values are in bold. Statistical trends in p value (p < 0.01) are underlined

*ANOVA* analysis of variance, *UHR* Ultra-High Risk for psychosis, *UHR*/*L* + UHR patients with language subjective disorder, *UHR/L-* UHR patients without language subjective disorder, *PANSS* Positive And Negative Syndrome Scale, *df* degrees of freedom, *F* F statistic value, *SOFAS* Social and Occupational Functioning Assessment Scale, *HoNOS* Health of the Nation Outcome Scale, *p* statistical significance, *η*^*2*^ partial eta squared, *EMM* Estimated Marginal Mean, *SE* Standard Error, *T0* baseline assessment, *T1* 1-year assessment time, *T2* 2-year assessment time

Bonferroni’s corrected p values are reported. Statistically significant p values are in bold. ^*^p < .01

## Discussion

To the best of our knowledge, this is the first study exploring the relationship between subjective language disturbances and clinical, functional, and outcome parameters in a sample of young UHR patients treated within an EIP service. Indeed, most studies on language impairment in UHR patients looked for automatic extraction of quantitative, biophysical (e.g., temporal/prosodic) markers or, in a specular way, FTDs, but exclusively from an objective/observational perspective. In our opinion, these objective disorders do not necessarily correspond to subjective, self-perceived distress, especially as specifically intended in the BS definition and theory [59]. Indeed, only when these neurobiological (“trans-phenomenal”) alterations reach sufficient clinical severity levels, they may first induce corresponding language disturbances that are primarily perceived as subjective pathological phenomena (i.e., BSs). Subsequently, language BSs, especially due to current relevant stressful events, may transit to the language clinical manifestations (and FTDs) behaviorally measurable with the PANSS [2, 3]. To explore this psychopathological evolution, future studies specifically examining the close relationship between biological/biophysical and the subjective language disturbances typically preceding objective FTDs of full-blown psychosis are thus needed, especially in order to support the hypothesis that language BSs are the first clinical features directly resulting from neurobiological language markers [60].

In this interesting perspective, the results of this examination showed a remarkable clinical link between UHR/L + patients and higher PANSS scores, especially in terms of negative symptoms and disorganization, both at baseline and across the follow-up. Overall, these findings suggest that the presence of subjective language disturbances at entry may be considered as an early clinical index of more severe and enduring psychopathology, despite the provision of specialized EIP interventions. Specifically, at baseline, these language BSs were notably associated with negative and disorganized features of psychosis (as behaviorally observed and measured by clinicians with the PANSS). This greater psychopathological severity at presentation was also confirmed in the UHR/L + subgroup by higher baseline prevalence rates of AP prescription and hospitalization, as well as by greater baseline PANSS scores in lack of judgment/insight.

Furthermore, although no inter-group differences were found in terms of DUI and 2-year incidence rates of service disengagement, new hospitalization, new suicidal thinking and behavior, psychosis transition, and symptoms remission across the follow-up, our mixed design ANOVA results drew two different longitudinal trajectories in the psychopathology of UHR/L + and UHR/L- participants, respectively. Indeed, at any point of the clinical course, the UHR/L + subgroup reported higher severity levels of psychopathology, independently by the provision of Pr-EP treatments (including AP medication). This poorer psychopathological outcome involved both affective and psychotic features (i.e., positive, negative, and disorganized dimensions), together with a poorer SOFAS functioning decline in UHR/L + participants across all time-points. This longitudinal functioning impairment in the UHR/L + subgroup specifically involved their social abilities (as indicated by higher HoNOS “Social problems” scores along the whole follow-up period) and appeared to start earlier (i.e., before the Pr-EP enrollment, as evidenced by their higher baseline HoNOS “Social problems” domain subscore and their greater baseline prevalence rate of work/school interruption).

As for *FTDs* in the UHR literature, some authors reported significant association of expressive communication disturbance (e.g., poverty of content, less referential cohesion) at baseline with poorer clinical status and lower socio-occupational functioning over time [61]. However, our outcome findings are not in line with the results of previous research. In this respect, contrary to the results of this investigation, an increase in frequency and duration of hospitalization was observed [62], as well as evidence that various expressive speech abnormalities (e.g., looseness, peculiar word/sentence usage, poverty of content, and weakening of goal) may predict psychosis transition [21, 63, 64]. Specifically, as for PANSS disorganized communication item, Spencer and colleagues [65] showed its significant correlation with an increased psychosis conversion hazard at baseline and persistent symptoms in illness trajectories over time. Overall, the vast majority of these studies concerned disturbance of expressive language but not receptive speech (i.e., language comprehension). However, considering only the expressive language disorders item, our outcome results did not change (see Supplementary Materials [Table S2] for details). Furthermore, contrary to what was reported in a recent network analysis on PANSS and SPI items [66], which showed no direct link between disturbance of expressive and of receptive speech and FTDs, we found a significant relationship between higher PANSS disorganization factor scores and the UHR/L + subgroup. This gives us the opportunity to hypothesize a more perceptible feature of language impairment during an important de-structuration of the language function.

In this respect, recent interesting studies exploring *objective speech markers* (especially, temporal and prosodic characteristics of language) found abnormalities in prosodic and temporal language functioning already in youths at UHR [67], i.e., before a full-blown psychosis onset. As for other evidence on objective language disorders in UHR populations, an investigation by Parola and co-workers [68] linked higher PANSS scores in the domain of disorganization with alterations in inference abilities in communicative intentions. According to the authors, this would imply a tendency to misattribute the communicative intentions of the speaker, due to increased pragmatism. These impaired inference abilities could also underlie a poor mentalization function (theory of mind), often observed in patients with FEP and frequently leading to symptoms related to the area of social cognition (which is primarily compromised especially in first-episode schizophrenia samples) [69]. However, also objective language markers substantially reflect expressive speech disturbance. Therefore, future studies specifically aimed at examining receptive language disorders (i.e., language comprehension) are strongly recommended.

Furthermore, such clinical aspects can seriously compromise the effectiveness of communication, resulting in an increase in negative social judgments [61]. From this perspective, some differences we detected whereby the UHR/L + subgroup could depend on this failure in the social domain and may induce less benefits from treatment in terms of global functioning (as measured with the SOFAS). This indicates the need for integrative support in social rehabilitation interventions taking into special account receptive and expressive language difficulties, also when exclusively confined in the individual subjectivity [70]. In this respect, NLP studies in UHR samples examining linguistic features linked to suicidal ideation found specific language disorders in whom expressed freely anger or other feeling, used firmly subject “I” at the top of the sentence, or stopped to use modal verbs [23, 71–73]. In these interesting investigations, crucial weakness was the risk of bias due to the high number of participants under AP treatment at baseline. However, the results of this examination support our evidence on the “univocity” of subjective language disturbances in UHR patients and their associations with poorer clinical and functional outcomes.

### Limitations

A first limitation of this investigation was related to the study design. Indeed, this was an observational research and data were collected not for specifically examining language disorders in UHR participants. Moreover, no specific instruments to analyzed language psychopathology or neurobiological markers were used. Finally, subjective language disturbances were not examined over time. Therefore, future longitudinal investigations including and correlating neurological, subjective and objective examinations on language impairment are needed.

Other crucial weaknesses included the relatively small sample size and the use of AP medications, which could affect language and formal thought functions. Therefore, future studies on larger and AP-naïve UHR samples also examining the role of AP drugs are needed. Specifically, we did not used AP treatment as a covariate in our analyses because no intergroup difference in terms of AP dosage at entry was observed (Table 1), despite a higher baseline prescription rate in the UHR/L + subgroup. Additionally, we did not consider psychosocial interventions as covariates because no between-group differences were found in terms of individual psychotherapy, family psychoeducation, and case management prevalence rates at entry (Table 1).

Furthermore, in this investigation we did not explore expressive and receptive language disturbances separately. Indeed, the subsample of UHR/L + participants exclusively with disorders of receptive speech was too small (only 11 patients). This did not allow us to conduct effective, separate statistical analyses. Therefore, as objective language measures were commonly concerned with speech production, future studies are needed that repeat our analyses for disturbances of expressive and of receptive speech separately in larger UHR samples. Finally, this was a single-center research that may not be considered representative of all current real-world UHR intervention protocols. Indeed, our findings are comparable only with investigations using similar study design and methodology.

## Conclusions

This investigation represents a first explorative analysis of subjective language disturbances in UHR patients. The results of this investigation overall showed the important role of language self-disorders as early subjective clinical indices of greater and more enduring severity in psychopathology of UHR patients, as well as of poorer clinical and functional outcomes over time. However, further studies are needed to explore this interesting field by using other assessment tools and other clinical endpoints, especially in order to evaluate the real predictive power of subjective language disorders for prognosis, especially transition to psychosis and suicidality.

## Supplementary Information

Below is the link to the electronic supplementary material.

Supplementary Material 1

## Data Availability

The data that support the findings of this research are available on reasonable request from the corresponding author. The data are not publicly available due to privacy or ethical restrictions.
