# Supplementary material for: Subjective language disturbances in young patients at ultra-high risk for psychosis (UHR): what relevance for clinical prognosis? A 2-year follow-up study
Source: Eur Arch Psychiatry Clin Neurosci. 2025 Aug 28;276(3):1097–110. doi: 10.1007/s00406-025-02094-w (PMC13002773; doi:10.1007/s00406-025-02094-w)
Supplement: Supplementary file 1 — Supplementary Material 1 [file 406_2025_2094_MOESM1_ESM.docx]

Table S1. Operative definitions of outcome parameters.

| -) Current suicidal ideation = BPRS item 4 score of ≥2.  -) Functional recovery = return to work/school.  -) SOFAS functional remission = SOFAS score of ≥60.  -) HoNOS functional remission = HoNOS item 9, 10, and 11scores of ≤2.  -) PANSS symptomatic remission = PANSS item P1, P2, P3, N1, N4, N6, G5, G9 scores of ≤ 3.  -) Service disengagement = complete lack of contact or untraceable for at least 3 months despite a need of treatment).  -) New suicide attempt = a potentially injurious, self-inflicted behavior without a fatal outcome for which there was (implicit or explicit) evidence of intent to die. |
| --- |

Note. BPRS = Brief Psychiatric Rating Scale; SOFAS = Social and Occupational Functioning Assessment Scale; HoNOS = Health of the Nation Outcome Scale; PANSS = Positive And Negative Syndrome Scale.

Figure S1. Profile plots: mixed ANOVA results on psychopathological and outcome parameters across the 2-year follow-up period in the two CHR-P subgroups.


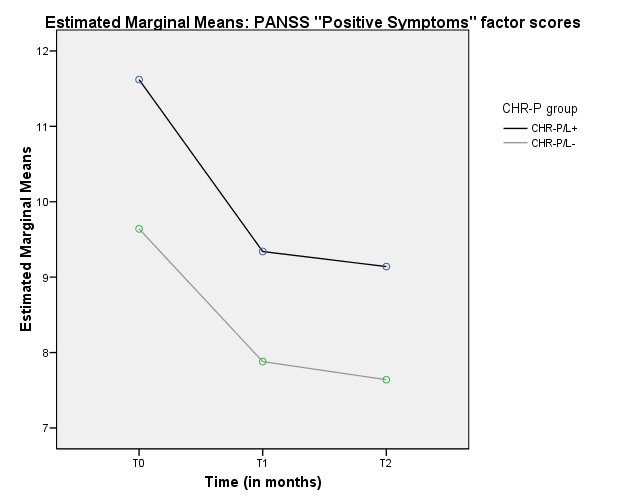


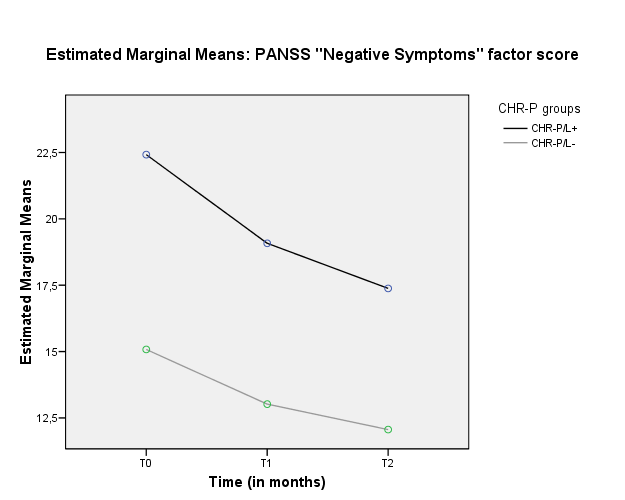


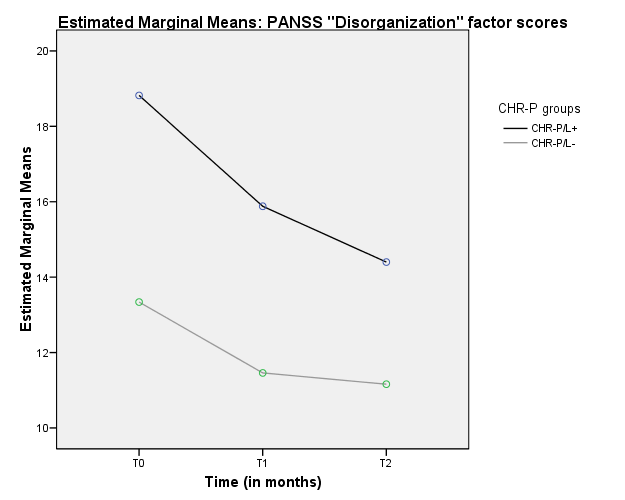


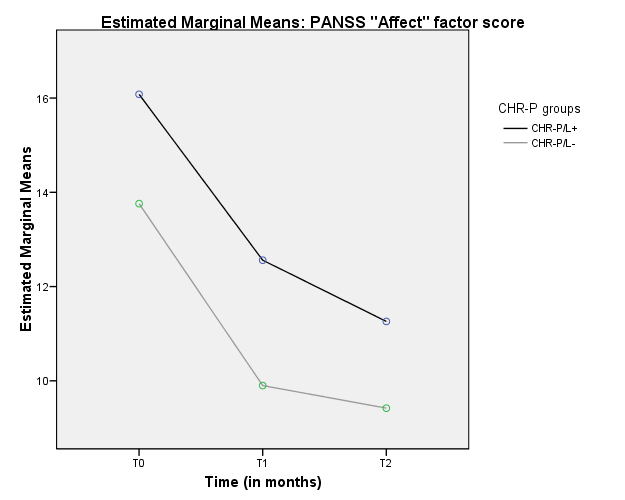


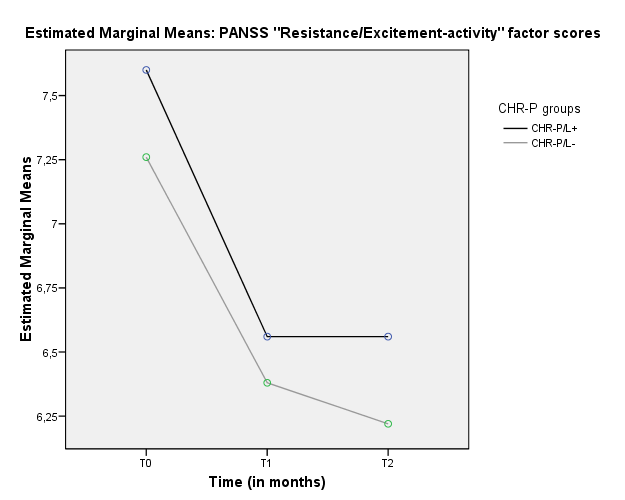


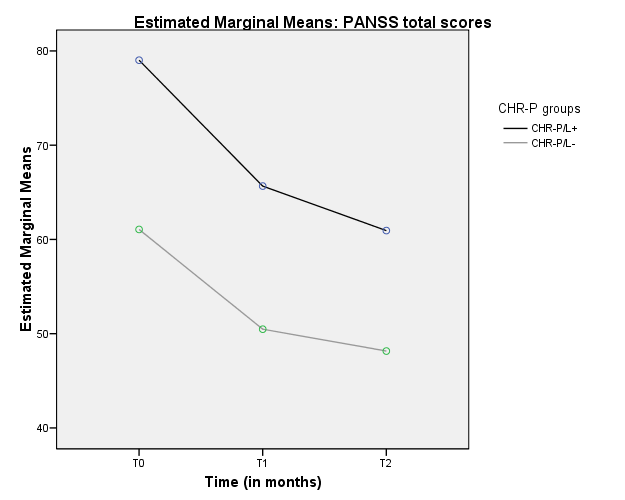


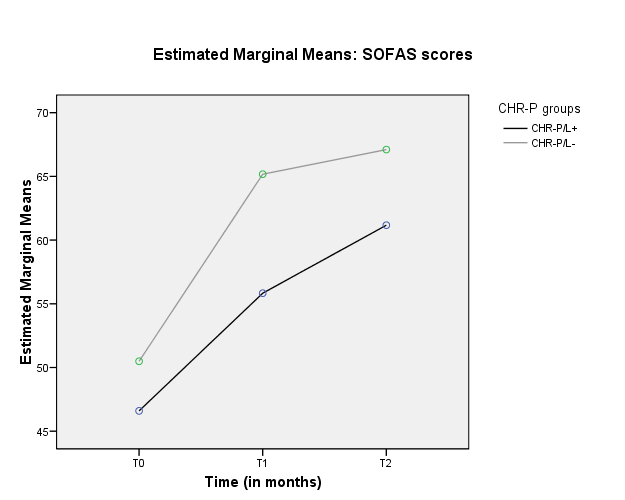


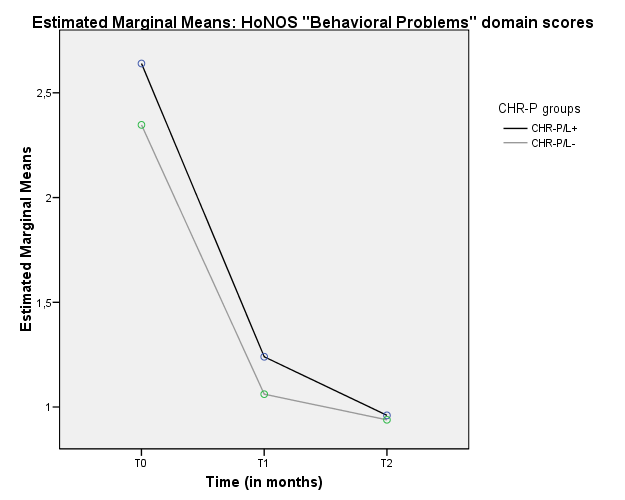


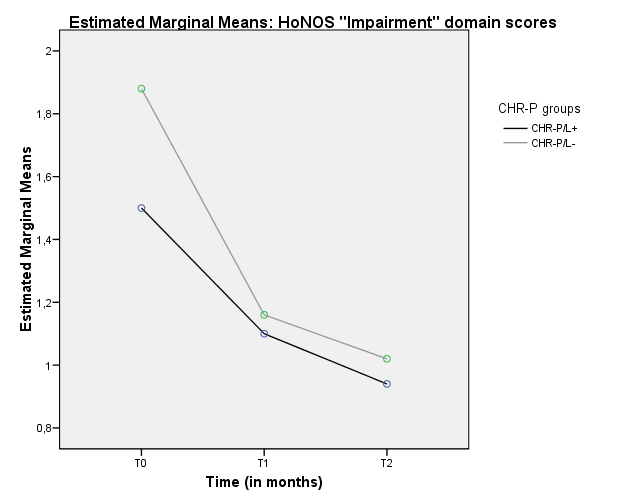


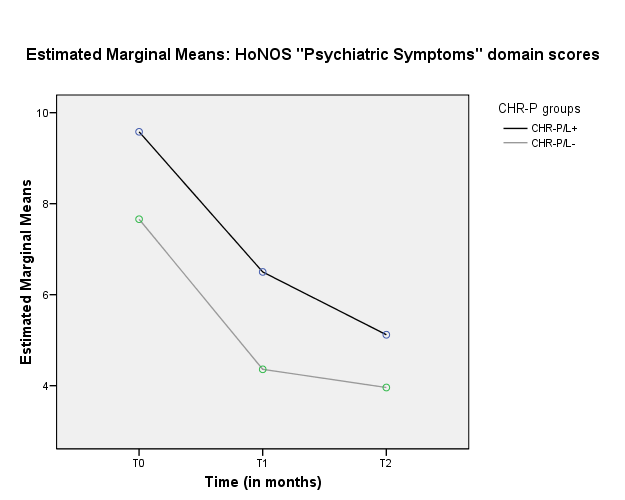


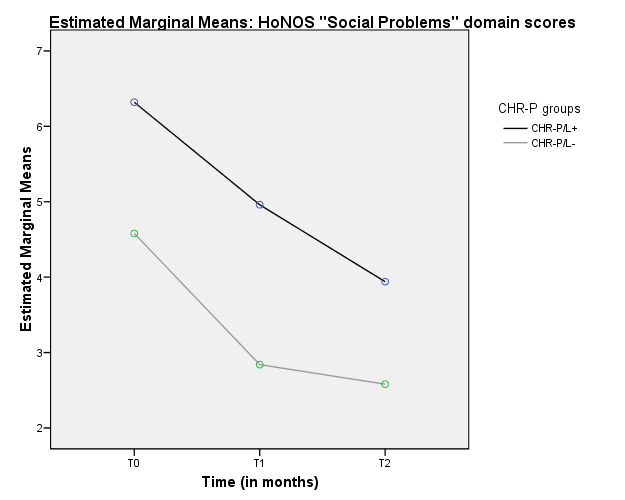


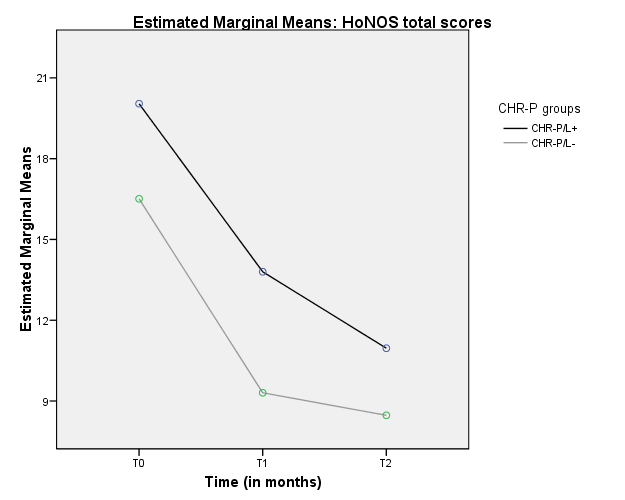


Note. ANOVA = analysis of variance; PANSS = Positive And Negative Syndrome Scale; CHR-P = Clinical High Risk for Psychosis; CHR-P/L+ = CHR-P individuals with language subjective disorder; CHR-P/L- = CHR-P individuals without language subjective disorder; SOFAS = Social and Occupational Functioning Assessment Scale; T0 = baseline assessment; T1 = 1-year assessment time; T2 = 2-year assessment time. As for PANSS and HoNOS scores, lower values indicate symptom/item improvement. As for SOFAS scores, higher values indicate functioning improvement.

Table S2. Kaplan-Meier survival analysis results: comparisons on 2-year time-to-event outcome incidence rate among the two UHR subgroups.

| UHR  subgroup | Number of events | cumulative proportion surviving at the time | | Mean (in months) for 2-year service disengagement incidence rate | | | |
| --- | --- | --- | --- | --- | --- | --- | --- |
|  |  | Estimate | SE | Estimate | SE | 95% CI | |
|  |  |  |  |  |  | Lower bound | Upper bound |
| UHR/EL+  UHR/EL-  (Overall) | 25  52  77 | 38.5%  49.5%  - | .060  .049  - | 18.923  17.962  18.329 | .831  .633  .505 | 17.295  16.722  17.340 | 20.552  19.202  19.319 |
| Log Rank (Mantel-Cox) | | | | Χ2 | df | p | |
|  |  |  |  | 1.445 | 1 | .229 | |
| UHR  subgroup | Number of events | cumulative proportion surviving at the time | | Mean (in months) for 2-year new hospitalization rate | | | |
|  |  | Estimate | SE | Estimate | SE | 95% CI | |
|  |  |  |  |  |  | Lower bound | Upper bound |
| UHR/L+  UHR/L-  (Overall) | 5  10  15 | 9.5%  11.3%  - | .040  .035  - | 23.186  22.887  23.000 | .439  .373  .275 | 22.326  22.156  22.461 | 24.047  23.617  23.539 |
| Log Rank (Mantel-Cox) | | | | Χ2 | df | p | |
|  |  |  |  | .215 | 1 | .643 | |
| UHR  subgroup | Number of events | cumulative proportion surviving at the time | | Mean (in months) for 2-year new suicide attempt incidence rate | | | |
|  |  | Estimate | SE | Estimate | SE | 95% CI | |
|  |  |  |  |  |  | Lower bound | Upper bound |
| UHR/L+  UHR/L-  (Overall) | 1  5  6 | 2.5%  6.1  - | .025  .028  - | 24.000  23.505  23.692 | .001  .271  .166 | 24.000  22.974  23.366 | 24.000  24.036  24.018 |
| Log Rank (Mantel-Cox) | | | | Χ2 | df | p | |
|  |  |  |  | 1.431 | 1 | .232 | |
| UHR  subgroup | Number of events | cumulative proportion surviving at the time | | Mean (in months) for 2-year psychosis transition rate | | | |
|  |  | Estimate | SE | Estimate | SE | 95% CI | |
|  |  |  |  |  |  | Lower bound | Upper bound |
| UHR/L+  UHR/L-  (Overall) | 7  10  17 | 12.7  12.8%  - | .045  .040  - | 22.780  23.134  23.000 | .510  .332  .274 | 21.780  22.483  22.464 | 23.779  23.785  23.536 |
| Log Rank (Mantel-Cox) | | | | Χ2 | df | p | |
|  |  |  |  | .044 | 1 | .833 | |

Note. UHR = Ultra-High Risk; UHR/EL+ = UHR individuals with expressive language subjective disorder (n=65); UHR/EL- = UHR individuals without language subjective disorder; SE = Standard Error; 95% CI = 95% Confidence Intervals; Log Rank = Logarithm Rank Test; X2= Chi-Square test; df = degrees of freedom; p = statistical value. Significant statistical p values are in bold.

**STROBE Statement**

|  | **Item**  **No** | | **Recommendation** |
| --- | --- | --- | --- |
| **Title and abstract** | 1 | | (*a*) we indicated the study’s design in both the title and the abstract |
|  |  | | (*b*) Provide in the abstract an informative and balanced summary of what was done  and what was found |
| **Introduction** |  | |  |
| Background/rationale | 2 | | We clearly explained the scientific background and rationale for our investigation: specifically, the fact that our study addressed a novel topic in the research of psychoses and language disorders. |
| Objectives | 3 | | We clearly stated specific objectives of our research at the end of the “Introduction” section. |
| **Methods** |  | |  |
| Study design | 4 | | We presented the main elements of study design (observational follow-up study) at the first part of the “Methods” section. |
| Setting | 5 | | We specifically described the setting and location (PARMS program within the Parma Department of Mental Health) of our investigation, including periods of recruitment, follow-up, and data collection. |
| Participants | 6 | | In the “methods” section (“Participants” paragraph) we gave the eligibility criteria and sources of selection of participants. We also described the methods of follow-up in the “Procedures” paragraph. |
| Variables | 7 | | In the “methods” section (“Procedures” and “Statistical Analysis” paragraphs), we  clearly defined all outcomes and predictors. We also gave diagnostic criteria for categorization. |
| Data sources/ measurement | 8* | | For each variable of interest, in the “Assessment” paragraph, we detailed methods of assessment (measurement). Moreover, in the “Statistical analysis” paragraph, we described methods of comparability between our subgroup. |
| Bias | 9 | | In the “Limitations” paragraph, we addressed potential sources of bias. |
| Study size | 10 | | In the “Methods” section, we described how the sample size was recruited. |
| Quantitative variables | 11 | | In the Methods” section, we explained qualitative and quantitative variables that were handled in the analyses. Moreover, we described how groupings were selected and why. |
| Statistical methods | 12 | | We describe all statistical methods. Specifically, we described the methods we used to examine subgroups. There were no missing data. Finally, we explained data about service disengagement. |
| **Results** |  | | |
| Participants | 13 | We reported numbers of individuals at each stage of our follow-up, previously examined for eligibility and subsequently included in the study. | |
| Descriptive data | 14 | We gave characteristics of study participants (i.e., demographic, clinical, social information). Specifically, we indicated number of participants. There were no missing data for the variables of our interest. | |
| Outcome data | 15 | We reported numbers of outcome events and measures over time | |
| Main results | 16 | We gave estimates and their precision (i.e., 95% confidence intervals). When relevant, we considered translating estimates in relative risk. | |
| **Discussion** |  |  | |
| Key results | 18 | We summarized key results with reference to study objectives. | |
| Limitations | 19 | We discussed limitations of the study in a specific paragraph of the “Discussion” section, taking into account sources of potential bias or imprecision. | |
| Interpretation | 20 | We gave a cautious overall interpretation of results considering objectives and limitations, results from similar studies, and other relevant evidence. | |

**Other information**

Funding 22 This investigation received no funds or grants.
